# Increased Circulating Th17 Cells after Transarterial Chemoembolization Correlate with Improved Survival in Stage III Hepatocellular Carcinoma: A Prospective Study

**DOI:** 10.1371/journal.pone.0060444

**Published:** 2013-04-02

**Authors:** Yuan Liao, Bo Wang, Zhi-Liang Huang, Ming Shi, Xing-Juan Yu, Limin Zheng, Shengping Li, Lian Li

**Affiliations:** 1 State Key Laboratory of Oncology in South China, Sun Yat-sen University Cancer Center, Guangzhou, P. R. China; 2 State Key Laboratory of Biocontrol, School of Life Sciences, Sun Yat-sen University, Guangzhou, P. R. China; 3 Department of Hepatobiliary Oncology, Sun Yat-sen University Cancer Center, Guangzhou, P. R. China; The University of Hong Kong, Hong Kong

## Abstract

Transarterial chemoembolization (TACE) has therapeutic effects in patients with unresectable hepatocellular carcinoma (HCC), but its impact on the cellular immune response during disease progression is largely unknown. Here we conducted a prospective study to evaluate the effect of TACE on immune status and to identify prognostic immune markers governing treatment success. In this study, 51 stage III HCC patients, 28 stage I HCC patients (TNM classification) and 20 healthy donors were enrolled. Flow cytometry and cytometric bead array were used to evaluate the circulating immune cell subsets, including CD4^+^ T cells (Th1, Th17 and Treg cells), CD8^+^ T cells, NK cells, and NKT cells, and plasma cytokines before TACE and 30 days after TACE. Interestingly, among those immune parameters, the frequency of circulating Th17 cells was higher in stage III HCC patients than in stage I HCC patients (*P* = 0.015) and healthy donors (*P*<0.001). Moreover, an increased frequency of circulating Th17 cells was observed 30 days after TACE (Th17*_D30_*) compared with the baseline level (*P* = 0.036). Kaplan-Meier analysis demonstrated that Th17*_D30_* was positively associated with overall survival (OS; *P* = 0.007) and time to progression (TTP; *P* = 0.009). Multivariate Cox analysis revealed that Th17*_D30_* was an independent prognostic factor for OS (HR = 0.317, *P* = 0.032) and TTP (HR = 0.304, *P* = 0.010). These results provide a potential prognostic marker for stage III HCC patients undergoing TACE and may be useful for identifying patients who can benefit from adjuvant immunotherapies.

## Introduction

Hepatocellular carcinoma (HCC) is among the most prevalent tumor types, with an increasing incidence and mortality worldwide [1], [2]. Despite improved diagnostic and treatment strategies, most HCC patients are diagnosed at advanced stages and are not eligible for potential curative therapies. Transarterial chemoembolization (TACE) is the most widely used treatment for unresectable HCC patients who have good liver function [3]. However, the therapeutic effects of TACE are not always satisfactory and involve many factors, such as appropriate patient selection, adequate therapy delivery and immune responses to TACE treatment [4]. Thus, evaluation of immune system modulation in HCC patients after TACE might elucidate the reasons for a favorable prognosis in only a subpopulation of patients.

HCC is usually derived from inflamed fibrotic and/or cirrhotic liver with intensive immune cell infiltration. Thus, the immune status may largely influence the biologic behavior of HCC [5], [6]. _ENREF_8Although research has focused on demonstrating the role of infiltrated immune cells in tumor tissues, an association of circulating immune cells and cytokines with disease progression has also been demonstrated. It has been reported that increased circulating regulatory T cells and IL-6 predict poor survival, while circulating NK cell activation is associated with improved survival in HCC patients [7], [8]. More recently, a novel IL-17-producing CD4^+^ T helper cell subset, termed Th17 cells, has been described. Th17 cells have potent pro-inflammatory properties and play an active role in inflammation and autoimmune diseases [9], [10]. However, the relationship between Th17 cells and tumor immunopathology has been controversial and highly depends on the context [11]. In our previous study, we observed that Th17 cells were highly enriched in the tumors of HCC patients and the levels of Th17 cells were positively correlated with poor survival [12]. In contrast, high levels of circulating Th17 cells were observed in long-term survivors of small cell lung cancer [13]. These findings indicate that the immune status may vary in different tumor microenvironments and have distinct effects on disease progression. However, little is known about the effect of the general immune status on disease progression in HCC patients undergoing TACE.

Immune markers predicting patient prognosis or response to therapy may advance the potential of personalized therapy in cancer treatment [14], [15]. In our latest study, we evaluated multiple subsets of infiltrating immune cells at tumor sites and identified several immune markers associated with recurrence in HCC patients who underwent curative resection [16]. Due to the heterogeneity of HCC and the diversity of treatment strategies, the patients' immune responses to TACE, and the significance of the immune response on prognosis may be somewhat complex. Therefore, we designed the current prospective study with three years of follow-up to evaluate the modulation and predictive utility of specific circulating immune cell subsets, including CD4^+^ T cells (Th1, Th17 and Treg cells), CD8^+^ T cells, NK cells and NKT cells, and plasma cytokines in unresectable HCC patients who underwent TACE.

## Materials and Methods

### Patients

This study protocol was approved by the Ethics Committee of the Sun Yat-sen University Cancer Center as stipulated by the Declaration of Helsinki, and written informed consent was obtained from all subjects. All of the samples were anonymously coded in accordance with local ethical guidelines.

From June 2009 to May 2010, a total of 1,196 patients were newly diagnosed with HCC in our institution, 329 underwent TACE and 161 met the inclusion criteria but 104 patients refused to take part in the study. In total, 57 patients were enrolled into this study. Six patients were excluded from this study because they were lost to follow-up. Finally, 51 patients with stage III HCC were included. The diagnosis of HCC was based on the diagnostic criteria for HCC used by the European Association for the Study of the Liver [17]. The clinical tumor stages were determined according to the TNM classification system of the International Union against Cancer (Sixth Edition). Stage III patients who met the following inclusion criteria were enrolled: 1) age between 18 and 75 years; 2) HCC with no previous treatment; 3) tumors with the largest diameter >5 cm; 4) Eastern Co-operative Group performance status of 0–1; and 5) normal liver or Child-Pugh A liver cirrhosis. Patients were excluded from the study if they had one or more of the following: 1) concurrent autoimmune disease, HIV or syphilis; 2) avascular or hypovascular tumor (defined as a tumor with all its parts less contrast-enhanced than the nontumorous liver parenchyma on arterial phase tomography scans); 3) diffuse-type HCC; 4) evidence of hepatic decompensation, including ascites, esophageal or gastric variceal bleeding, or hepatic encephalopathy; 5) severe underlying cardiac or renal disease; 6) portal vein tumor thrombosis with complete main portal vein occlusion and without adequate collateral circulation around the occluded portal vein; or 7) clinical symptoms or signs of sepsis.

All 51 patients with stage III HCC received TACE treatment (Group 1) and 28 patients with stage I HCC who underwent surgical resection or radiofrequency ablation were included as the control group (Group 2). Patients did not receive any other treatment within 30 days after TACE. Control blood samples were obtained from 20 healthy donors attending the Guangzhou Blood Center (Group 3) and all of the controls were negative for hepatitis B, hepatitis C, HIV and syphilis. The clinicopathologic characteristics of all patients were summarized in Table 1.

**Table 1:** Patient characteristics.

Variables	^a^ Group 1	^b^ Group 2
Cases (n)	51	28
Age, years (median, range)	52, 26–75	48, 32–69
Gender (male/female)	46/5	27/1
HBsAg (negative/positive)	4/47	2/26
Cirrhosis (absent/present)	4/47	4/24
ALT, U/L (median, range)	44.3, 14–160.8	32.1, 14.3–58.2
AFP, ng/mL (median, range)	1585, 1.8–129470	158.6, 2.2–7492
Tumor size, cm (median, range)	10.6, 5–19	2.8, 1.3–4.8
Tumor multiplicity (solitary/multiple)	21/30	28/0
Vascular invasion (absent/present)	25/26	28/0
TNM stage	III	I

Note: ^a^ Group 1: Stage III HCC patients who underwent TACE; ^b^ Group 2: Stage I HCC patients who underwent surgical resection or radiofrequency ablation.

Abbreviations: HBsAg, hepatitis B surface antigen; ALT, alanine aminotransferase; AFP, alpha-fetoprotein.

### Transarterial chemoembolization procedure

Transarterial chemoembolization treatment was performed following the techniques described previously [18], [19]. A selective 5-French catheter was introduced and visceral angiography was carried out to assess the arterial blood supply to the liver and to confirm patency of the portal vein. Depending on the size, location and arterial supply of the tumor, the tip of the catheter was advanced into the right or left hepatic artery, or into the tumor-feeding branches. After safe positioning of the catheter, the emulsion of lipiodol and chemotherapeutic agents was infused. The treatment regimen was comprised of carboplatin 300 mg [20], [21], [22] (Bristol-Myers Squibb, New York, USA), epirubicin 50 mg (Pharmorubicin, Pfizer, USA), and mitomycin C 8 mg (Zhejiang Hisun Pharmaceutical Co. Ltd., Taizhou, China) mixed with 5 mL ethiodized oil (Lipiodol Ultra-Fluide; Andre' Guerbet Laboratories, Aulnay-Sous-Bois, France). Subsequently, embolization was performed with injection of absorbable gelatin sponge particles (1–2 mm in diameter, Gelfoam; Hanzhou alc Ltd, Hangzhou, China) through the catheter to reach stasis in the tumor-feeding artery. The treatment regimen was used consistently in this study, regardless of tumor number and size. Patients were observed carefully after treatment and analgesia was given if necessary.

### Follow-up

All of the stage III HCC patients were closely followed until June 15, 2012. The median duration of follow-up for the stage III patients was 12.7 months (range 1.0–34.8 months). The routine follow-up program included a serum alpha-fetoprotein (AFP) assay, abdominal ultrasonography, and liver function tests at intervals of one month for the first year and every three months thereafter [18]. Patients underwent abdominal computed tomography (CT) scans during the first month after TACE, and liver CT scans were performed at three-month intervals during the first year post-treatment. A routine CT scan was performed every six months thereafter. Extrahepatic organ examination was also carried out if patients had extrahepatic metastases. Liver magnetic resonance imaging was also used to define suspicious lesions demonstrated on CT and/or by elevated AFP. The Response Evaluation Criteria in Solid Tumors (RECIST) was used to measure tumor response: CR (complete response), disappearance of all target lesions; PR (partial response), at least a 30% decrease in the sum of the longest diameter of the target lesions; SD (stable disease), neither PR nor progressive disease; PD (progressive disease), at least a 20% increase in the sum of the longest diameter of the target lesions, or the appearance of new lesions or metastasis [23]. Radiological staging after treatment was based on the RECIST. Overall survival (OS) was defined as the interval between treatment and death or last observation of surviving patients. Time to progression (TTP) was defined as the interval between treatment and disease progression or last observation of patients without disease progression. Another session of TACE was performed every 4–10 weeks until CT scans and AFP levels suggested stabilization of the tumor, or until it was not technically feasible either because of hepatic artery occlusion or impaired liver function.

### Blood collection and peripheral blood mononuclear cell isolation

Blood samples from stage III HCC patients were withdrawn immediately before treatment (D_0_) and 30 days after treatment (D_30_). Blood samples from 20 healthy donors and 28 stage I HCC patients before treatment were analyzed as controls. All the 51 stage III HCC patients were assessed before treatment and 30 patients returned for the immunological follow-up 30 days after treatment. For phenotype analysis, peripheral blood mononuclear cells (PBMC) were isolated from fresh heparinized blood by Ficoll density gradient centrifugation [24]. An aliquot of plasma was separated from each blood specimen and stored at −80°C for subsequent analysis.

### Flow cytometric analysis

PBMC were stained with the following antibodies: allophycocyanin (APC)-conjugated anti-CD3, phycoerythrin-Texas Red (ECD)-conjugated anti-CD3, fluorescein isothiocyanate (FITC)-conjugated anti-CD4, phycoerythrin-cyanin 7 (PE-Cy7)-conjugated anti-CD4, FITC-conjugated anti-CD8, phycoerythrin (PE)-conjugated anti-CD8, PE-conjugated anti-IFN-γ (Beckman Coulter, Fullerton, ST, USA), FITC-conjugated anti-CD3-FITC, PE-Cy7- conjugated anti-CD56 (BD Biosciences, San Jose, CA, USA), Alexa Fluor 647 (AF647)-conjugated anti-IL-17A, APC-conjugated anti-FoxP3 and PE-conjugated anti-Perforin (eBioscience, San Diego, CA, USA). For intracellular cytokine detection, cells were stimulated for 5 h with Leukocyte Activation Cocktail (BD Biosciences), stained with surface markers, fixed and permeabilized with IntraPre Reagent (Beckman Coulter), and then stained with anti-IL-17A and anti-IFN-γ. For FoxP3 analysis, the APC anti-FoxP3 Staining Set (eBioscience) was used according to the manufacturer's instructions. Data were acquired using a Cytomics™ FC500 (Beckman Coulter) and analyzed with CXP analysis software (Beckman Coulter).

### Cytokine analysis

Plasma cytokine levels of IL-2, IL-4, IL-6, IL-10, IL-17A, IFN-γ, and TNF-α were evaluated using the human Th1/Th2/Th17 cytokine Cytometric Bead Array (CBA) Kit (BD Biosciences) according to the manufacturer's instructions. Fluorescence was detected using a Cytomics™ FC500 (Beckman Coulter), and the data were analyzed using CBA analysis software (BD Biosciences). The assay sensitivities for IL-2, IL-4, IL-6, IL-10, IL-17A, IFN-γ, and TNF-α were 2.6, 4.9, 2.4, 4.5, 18.9, 3.7 and 3.8 pg/mL, respectively.

### Statistical analysis

Data analysis was performed using SPSS 13.0 software (SPSS Inc., Chicago, IL, USA), and the data were expressed as the mean ± SD for continuous variables. The statistical significance of the difference between two groups was determined using the non-parametric Mann-Whitney *U*-test. The Wilcoxon signed-rank test was used for paired samples. Correlations between variables were determined by Spearman's rho tests. The Kaplan-Meier method was applied to assess actuarial survival using median value as a cut-off to separate patients into a high and low group. The log-rank test was applied to compare the groups. Univariate analysis was performed to assess significant clinicopathological and immunological parameters that influence overall survival and time to progression after treatment. A multivariate analysis was performed by Cox regression for significant variables in the univariate analysis. *P* value of less than 0.05 was considered significant.

## Results

### High frequency of Th17 cells in stage III HCC patients

**Figure 1:**
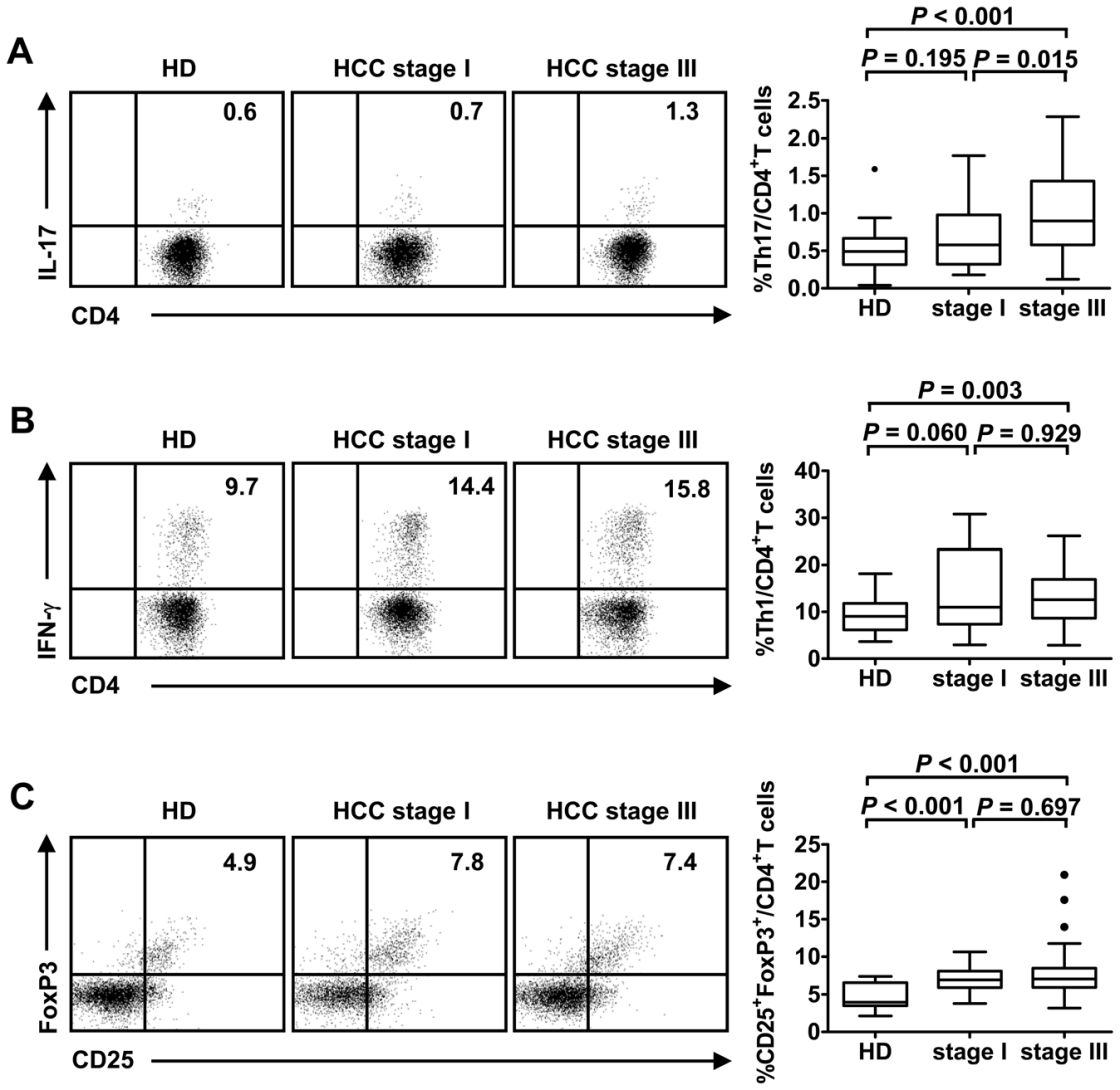
The frequencies of T cell subsets in peripheral blood from HCC patients and healthy donors. Representative flow cytometry data and statistical analyses were shown comparing the baseline frequencies of circulating Th17 (A), Th1 (B) or Treg (C) cells between patients with stage III HCC (n = 51), patients with stage I HCC (n = 28) and healthy donors (n = 20). (A) Th17 cells were gated from the IL-17^+^ subsets of the CD3^+^CD4^+^ T cells. (B) Th1 cells were gated from IFN-γ^+^ subsets of CD3^+^CD4^+^ T cells. (C) Treg cells were gated from CD25^+^FoxP3^+^ subsets of CD3^+^CD4^+^ T cells. The data are expressed as box plots, in which the horizontal lines illustrate the 25^th^, 50^th^, and 75^th^ percentiles. HD, healthy donors.

To determine the general immune status of HCC patients, we used flow cytometry to measure the frequencies of various immune cell subsets, including CD4^+^ T cells (Th1, Th17, and Treg cells), CD8^+^ T cells, NK cells, and NKT cells, and cytokine levels (IL-2, IL-4, IL-6, IL-10, IL-17A, IFN-γ and TNF-α) in peripheral blood from 51 stage III HCC patients, 28 stage I HCC patients before treatment and 20 healthy donors. In general, the frequencies of total T cells, CD4^+^ T cells, CD8^+^ T cells, NK cells and NKT cells in HCC patients did not differ significantly from healthy donors (Table S1). Subsequently, we compared different CD4^+^ T cell subsets and found that the frequencies of IL-17-producing CD4^+^ T (Th17) cells, IFN-γ-producing CD4^+^ T (Th1) cells and CD4^+^CD25^+^FoxP3^+^ cells (Treg) were all increased in stage III HCC patients compared with healthy donors (Figure 1). However, when we compared HCC patients with different tumor stages, only the frequency of Th17 cells was higher in stage III HCC patients compared with stage I HCC patients (stage III, 1.0% ± 0.6% vs. stage I, 0.7% ± 0.4%, *P* = 0.015; Figure 1A). These results indicate that the number of Th17 cells increases with HCC development, and that Th17 cells may play a more important role in advanced stage HCC patients. In addition, among the seven cytokines determined in the plasma, IL-6 was significantly higher in patients with stage III HCC patients than stage I HCC patients (*P*<0.001), whereas the other cytokines were below the level of sensitivity for detection of the CBA kit in most samples (Table S2).

### Circulating Th17 cells are increased in stage III HCC patients after TACE

**Figure 2:**
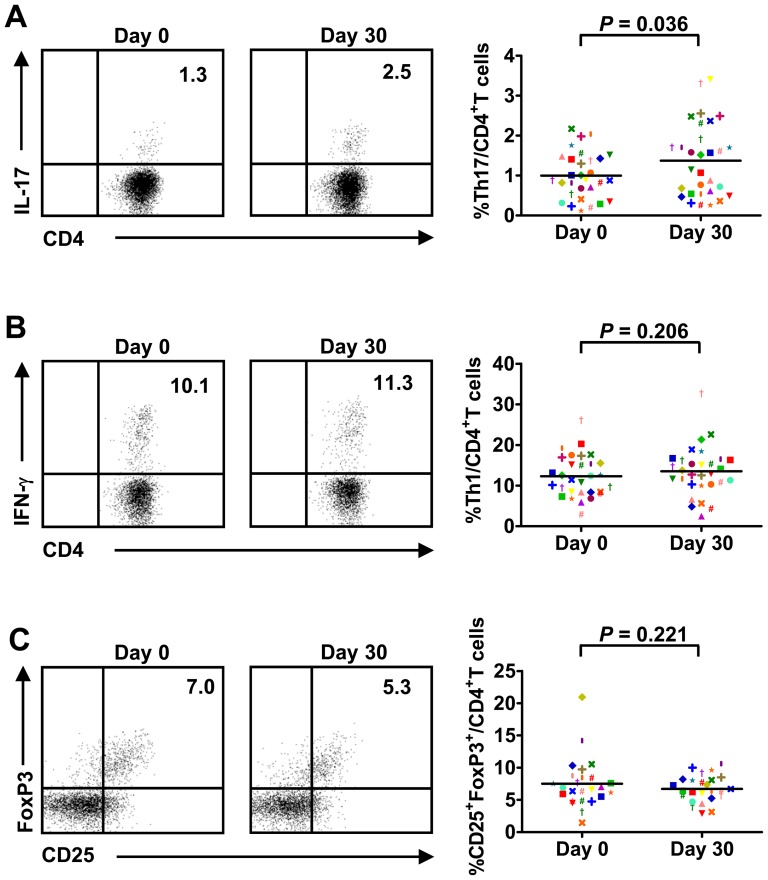
Significant increase of circulating Th17 cells after TACE. The frequencies of circulating T cell subsets were analyzed in HCC patients the day before TACE (D_0_) and 30 days after TACE (D_30_). Representative flow cytometry data and statistical analysis demonstrating that the frequency of Th17 cells (A) was increased 30 days after TACE while the frequencies of Th1 (B) and Treg (C) cells were not altered. The mean values and significant differences are shown in each panel.

Previous studies have shown that TACE induces necrosis and causes inflammation with cytokine production, which may favor immune activation and presentation of tumor-specific antigens [4]. To assess whether TACE alters the homeostasis of lymphocyte populations circulating in peripheral blood, the frequencies of various lymphocyte subsets were evaluated in blood samples drawn from stage III HCC patients the day before TACE (D_0_) and 30 days after TACE (D_30_) (Table S3). A significant increase in the frequency of Th17 cells was observed at D_30_ compared with D_0_ in these patients (Figure 2A), whereas the frequency of Th1 cells was not altered after treatment (Figure 2B). In addition, TACE had little effect on the Treg frequency in peripheral blood (Figure 2C). Further analysis of plasma cytokines revealed that IL-6 levels were upregulated soon after TACE treatment but returned to baseline levels at D_30_ (data not shown). Taken together, our data indicate that TACE may have a long-term effect on the Th17 cell population and augment its frequency in peripheral blood.

### Increased circulating Th17 cell frequency one month after TACE predicts improved survival

**Figure 3:**
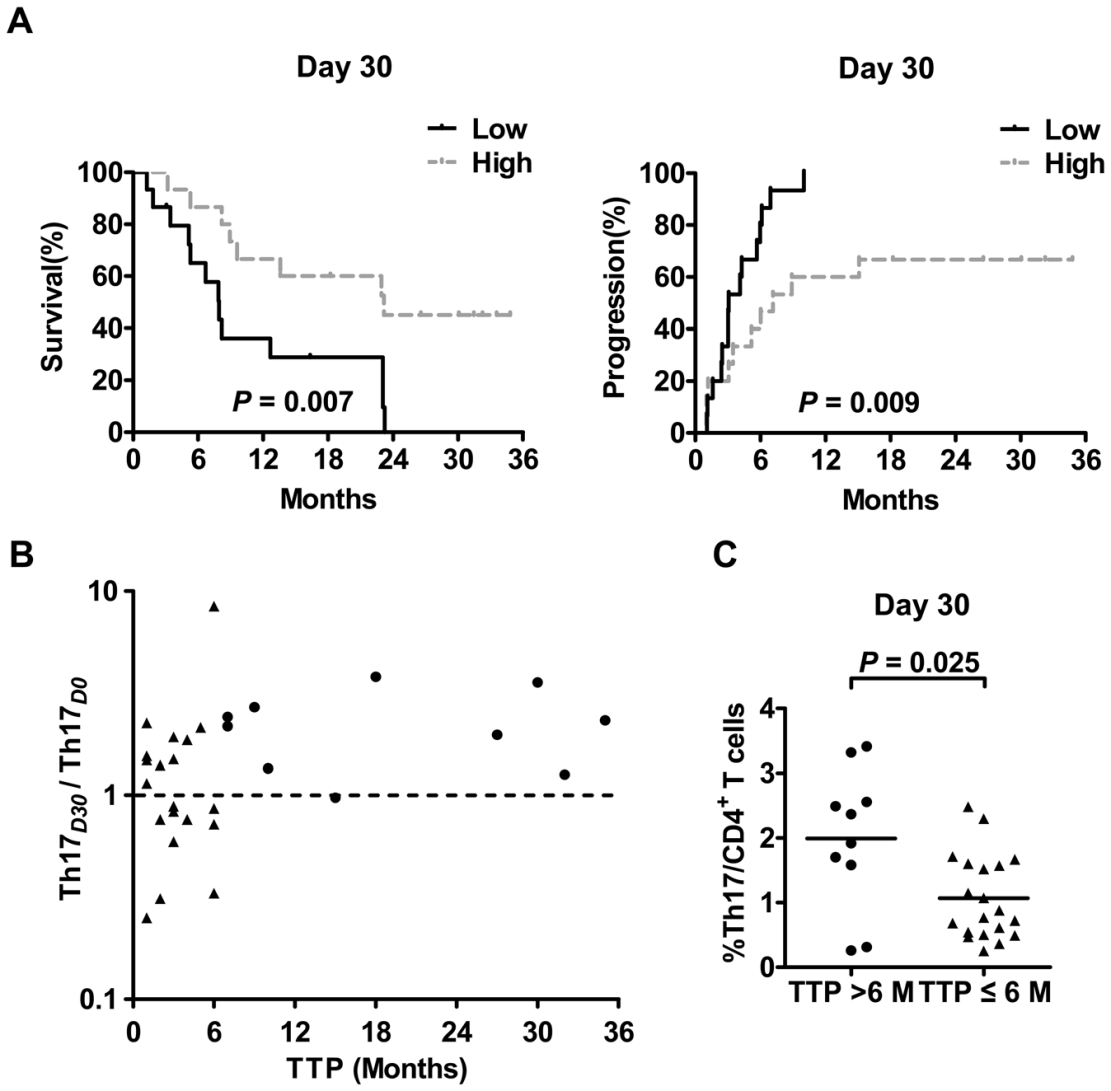
Increased frequency of circulating Th17 cells after TACE predicted improved survival. (A) Individuals in the high Th17 frequency group had significantly improved survival compared with those in the low Th17 frequency group 30 days after TACE. The samples were divided into two groups by the median Th17 frequency. Overall survival (OS) and time to progression (TTP) were estimated by the Kaplan-Meier method and compared using the long-rank test. (B) The association between the percentual change of Th17 levels (Th17_D30_/Th17_D0_) and TTP. Th17*_D0_*, the percentage of Th17 cells in CD4^+^ T cells before TACE; Th17*_D30_*, the percentage of Th17 cells in CD4^+^ T cells 30 days after TACE. The vertical axis is displayed on a logarithmic scale (base 10). (C) Patients with extreme good prognosis (TTP>6 months, circles) had higher Th*_D30_* than the other patients (TTP ≤ 6 months, triangles). M, months.

Since circulating Th17 cells were significantly increased after TACE in stage III HCC patients, we next investigated the association of Th17 cells with the survival in those patients. We analyzed relevant clinical information and correlated the data with the frequencies of circulating Th17 cells at D_0_ (Th17*_D0_*) and D_30_ (Th17*_D30_*). Kaplan-Meier analysis revealed that Th17*_D30_* rather than Th17*_D0_* (Figure 3A and Figure S1) was positively associated with OS (*P* = 0.007) and TTP (*P* = 0.009). Patients with higher Th17*_D30_* had significantly longer OS (median: 22.9 months) or TTP (median: 7.2 months) than patients with lower Th17*_D30_* (median: OS: 7.9 months; TTP: 3.1 months). Moreover, five of the six patients who did not experience disease progression within one year after TACE were in the high Th17*_D30_* group.

Additionally, the increase of Th17 cells from D_0_ to D_30_ only occurred in a part of the patients (63%, 19 of 30). Then, we evaluated the prognostic value of the change of Th17 levels represented by Th17*_D30_*/Th17*_D0_* in univariate Cox regression for OS and TTP. The results showed that it could not yet be considered as a prognostic variable probably due to the limited number of patients (Table 2). However, there was a positive correlation between the percentual change of Th17 levels (Th17_D30_/Th17_D0_) and TTP (r = 0.426, *P* = 0.019, Figure3B). The progression of HCC after TACE is more likely to develop within the first 6 months [25]. Among the 30 patients, 1/3 of them were free of progression within 6 months. Then we further analyzed the change of Th17 levels in those patients with extremely good prognosis. As shown in Figure 3B, 9 of the 10 patients with extremely good prognosis (TTP>6 months) had increased Th17*_D30_* levels. Meanwhile 10 of the 11 patients with decreased Th17*_D30_* levels had relatively poor prognosis (TTP ≤ 6 months). In addition, the ten patients with extreme good prognosis also had higher Th*_D30_* than the other patients (Figure 3C).

**Table 2:** Univariate and multivariate analysis of factors associated with the survival and progression of stage III HCC patients who underwent TACE (n = 30).

Variables	OS				TTP			
	Univariate *P*	Multivariate			Univariate *P*	Multivariate		
		HR	95% CI	*P*		HR	95% CI	*P*
Age(>55 vs. ≤55 years)	0.419			NA	0.872			NA
Gender (male vs. female)	0.173			NA	0.311			NA
HBsAg (positive vs. negative)	0.506			NA	0.568			NA
Cirrhosis (present vs. absent)	0.268			NA	0.454			NA
AFP (>400vs. ≤400 ng/mL)	0.111			NA	0.150			NA
LDH (>245 vs. ≤245 U/L)	**0.037**	2.630	0.759–9.112	0.127	0.314			NA
INR (>1.06 vs. ≤1.06)	**0.017**	0.822	0.233–2.904	0.760	0.369			NA
Tumor number (multiple vs. solitary)	0.515			NA	0.574			NA
Tumor size (>10 vs. ≤10 cm)	0.471			NA	0.993			NA
Vascular invasion (present vs. absent)	**0.015**	3.566	1.382–9.198	**0.009**	0.745			NA
Radiological stage at D_30_ (SD+PD vs. CR+PR)	0.104			NA	**0.018**	3.294	1.279–8.484	**0.014**
Th17*_D0_* (high vs. low)	0.944			NA	0.953			NA
Th17*_D30_* (high vs. low)	**0.011**	0.317	0.111–0.904	**0.032**	**0.012**	0.304	0.123–0.752	**0.010**
Th17*_D30_*/Th17*_D0_* (>1 vs. ≤1)	0.534			NA	0.080			NA

Abbreviations: OS, overall survival; TTP: time to progression; HR: hazard ratio; CI: confidence interval; HBsAg, hepatitis B surface antigen; AFP, alpha-fetoprotein; LDH, lactate dehydrogenase; INR, international normalized ratio; SD, stable disease; PD, progressive disease; CR, complete response; PR, partial response; D_30_, 30 days after TACE; Th17*_D0_*, the percentage of Th17 cells in CD4^+^ T cells before TACE; Th17*_D30_*, the percentage of Th17 cells in CD4^+^ T cells 30 days after TACE; NA, not applicable.

In univariate analysis (Table 2), Th17*_D30_*, lactate dehydrogenase, international normalized ratio and vascular invasion were associated with OS. For TTP, only Th17*_D30_* and radiological stage at D*_30_* had prognostic significance. Multivariate Cox proportional hazards analysis was performed, and variables that were associated with survival by univariate analysis were adopted as covariates. The multivariate analysis revealed that Th17*_D30_* was an independent prognostic factor for both OS (HR = 0.317, *P* = 0.032) and TTP (HR = 0.304, *P* = 0.010) in stage III HCC patients.

### Th17 cells are positively correlated with effector T cell subsets

**Figure 4:**
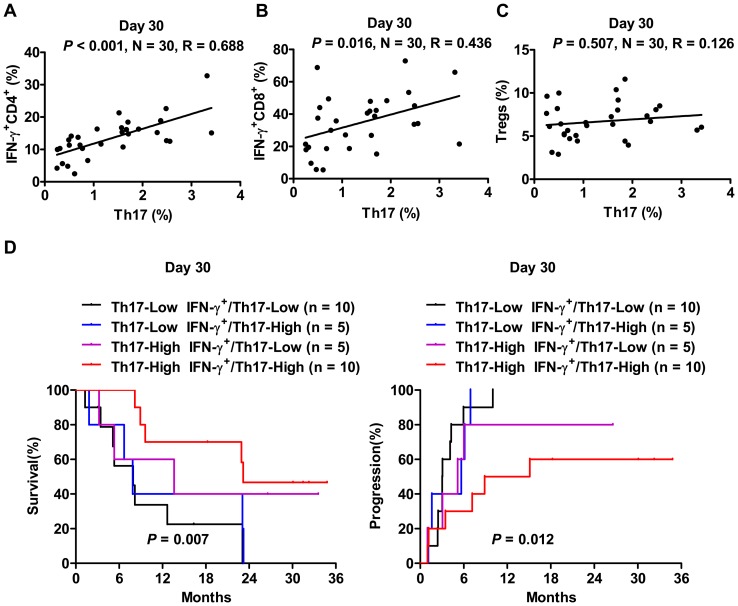
Th17 cells and their association with effector T cell subsets. (A–C) The correlations between the frequencies of circulating Th17 cells and IFN-γ^+^CD4^+^ T cells (A), IFN-γ^+^CD8^+^ T cells (B), and Treg (C) cells were evaluated. (D) OS and TTP of HCC patients according to the frequencies of Th17 cells in combination with IFN-γ^+^IL-17^+^CD4^+^ T cells 30 days after TACE. The TTP and OS of patients with Th17-Low IFN-γ^+^ IL-17^+^-Low are shown in black, Th17-Low IFN-γ^+^IL-17^+^-High in blue, Th17-High IFN-γ^+^ IL-17^+^-Low in purple and Th17-High IFN-γ^+^IL-17^+^-High in red. The log-rank *P* values for TTP and OS were calculated by comparing patients with Th17-High IFN-γ^+^ IL-17^+^-High with patients with Th17-Low IFN-γ^+^ IL-17^+^-Low.

The antitumor effects of Th17 cells are thought to be mediated by the recruitment of cytotoxic effector cells [11]. Thus, we evaluated the correlation between Th17 cells and other lymphocyte subsets. Pairwise comparisons were performed by measuring Spearman correlation coefficients (r). We found that Th17 cells were positively correlated with IFN-γ-expressing effector T cell subsets, including IFN-γ^+^CD4^+^ T cells (r = 0.688, *P*<0.001, Figure 4A) and IFN-γ^+^CD8^+^ T (Tc1) cells (r = 0.436, *P* = 0.016, Figure 4B), at D_30_ after TACE. In contrast, there was no quantitative correlation between Th17 and Treg cells (Figure 4C).

In accordance with our previous studies [12], [26], approximately 12% of the circulating Th17 cells expressed IL-17 and IFN-γ (data not shown). Thus, we further divided patients into four groups by combining the high and low groups of both frequencies of Th17 and IFN-γ^+^IL-17^+^CD4^+^ T cells at D_30_ after TACE. Kaplan-Meier curves showed that the best combination associated with prolonged OS and TTP was Th17-High and IFN-γ^+^IL-17^+^-High group (Figure 4D).

## Discussion

TACE is currently considered as the standard of care for unresectable HCC patients, and the immune status may significantly influence the progression and clinical outcome of HCC. Since it is not practical to determine the immune status at the tumor site in unresectable HCC patients, and many tumor-infiltrating immune cells are derived from peripheral blood, we investigated the levels and clinical significance of circulating immune cell subsets and plasma cytokines in these patients before and after TACE. The results demonstrated that the frequency of Th17 cells increased 30 days after TACE compared with the baseline level, and that this high frequency of Th17 cells was associated with favorable clinical outcome and may represent a potential prognostic marker for stage III HCC patients undergoing TACE.

Th17 cells have been detected in the blood and tissues of patients with various cancers, but their contribution to tumor pathogenesis remains elusive. Adoptive transfer of tumor-specific Th17-polarized cells can induce tumor eradication [27], [28], whereas Th17 cells can promote angiogenesis and increase neutrophil recruitment, which in turn exert protumorigenic activity [29], [30]. It is possible that Th17 cells may play distinct roles in various tumor environments and different stages of tumor development. In the current study, we provided evidence showing that the high frequency of Th17 cells one month after TACE was associated with prolonged OS and TPP in stage III HCC patients. Although human Th17 cells have no direct cytotoxic effect on cancer cells, Th17 cell-derived IL-17 and IFN-γ synergistically induce CXCL9 and CXC10 production in ovarian cancer, which in turn leads to recruitment of Th1-type effector T cells [31]. In accordance with these results, a positive correlation was observed between Th17 and IFN-γ^+^ effector T cells in our study. When we combined the frequencies of Th17 cells with IFN-γ^+^IL-17^+^ cells for prognostic analyses, the best combination associated with prolonged TTP and OS was the Th17-High and IFN-γ^+^IL-17^+^-High group. Moreover, recent studies demonstrated that Th17 cells are long-lived effector memory cells that are resistant to apoptosis and induce enhanced eradication of established tumors compared with their Th1 counterparts [32], [33]. Taken together, these findings suggest that the high frequency of Th17 cells one month after TACE may reflect long-lasting antitumor immune responses to TACE, which may play an important role in preventing disease progression in stage III HCC patients.

The precise mechanism underlying the increased frequency of circulating Th17 cells in HCC patients with advanced stages is currently unclear. The sources of circulating Th17 cells may include the differentiation of Th17 cells from CD4^+^ T cells and the entry of tumor-infiltrating Th17 cells into the circulation. It has been shown that high levels of IL-6 are often found in advanced tumors and are involved in the differentiation of Th17 cells [34], [35]. In one of our latest studies [26], we demonstrated that IL-6 effectively increased the frequency of Th17 cells in vitro. Here we found that the levels of IL-6 were significantly elevated in the plasma of stage III HCC patients compared with healthy donors and stage I HCC patients, which may partially account for the high frequency of circulating Th17 cells in advanced HCC patients. In addition, Th17 cells could expand in HCC tissue and exit into the circulation along with vascular invasion and metastasis in HCC patients with advanced stages [12], [26].

The increased frequency of Th17 cells observed after TACE may partly be attributed to tumor necrosis caused by the treatment, which could induce antitumor immune responses. Previous studies have demonstrated that TACE may favor the presentation of tumor-specific antigens to antigen-presenting cells [4]._ENREF_4 Tumor-activated monocytes secrete a set of key pro-inflammatory cytokines that trigger the proliferation of functional Th17 cells [26]. In another study, we observed higher numbers of intratumoral IL-17-producing cells in HCC patients who underwent TACE before surgical resection compared to those who did not receive any treatment prior to surgery (unpublished results). Moreover, recent studies demonstrated that Th17 cells were resistant to chemotherapy-mediated cell death, and cyclophosphamide was able to promote Th17 cell differentiation [32], [33], [36]. Thus, chemotherapeutic agents that are used to destroy tumor tissue during TACE may also contribute to the increased frequency of Th17 cells.

IFN-γ-producing Th1 cells play an important role in antitumor immunity [37]. However, circulating Th1 cells were not correlated with disease progression or survival in our study (data not shown). One possible explanation is that most of the HCC samples in our study were derived from chronic inflammation induced by hepatitis B virus infection accompanied by a vigorous cytotoxic and helper T cell response [38]. Another reason may be that tumor-specific effector cells, which are able to recognize tumor-specific antigens and induce cytotoxicity against tumor cells, only account for a small proportion of Th1 cells in PBMC. In contrast, though tumor-specific Th17 cells only remain a minor population in PBMC, they mediate destruction of advanced tumors better than their Th1 counterparts [28]. Thus, detecting and enhancing tumor-specific immune responses may provide a more efficient way to improve survival in advance HCC.

In this prospective study, we have demonstrated that a high frequency of Th17 cells post-treatment is associated with improved prognosis in stage III HCC patients undergoing TACE, suggesting an important role for Th17 cells in antitumor immunity. These data indicate that reconstitution of the Th17 pool may provide a promising adjuvant immunotherapeutic strategy for stage III HCC patients undergoing TACE. Furthermore, screening for Th17 cells in peripheral blood provides a noninvasive, straightforward prognostic marker, which may also be useful for determining which patients may benefit from adjuvant immunotherapies [39], [40], [41].

## Supporting Information

Figure S1 **Prognostic significance of Th17 cells in HCC patients before TACE.** Kaplan-Meier curve for overall survival (A) and time to progression (B) by the frequency of Th17 cells before TACE (n = 51).
(TIF)

Table S1 **Comparison of the frequencies of various lymphocyte subsets in healthy donors and HCC patients.**
(DOC)

Table S2 **Plasma levels of Th1, Th2 and Th17 associated cytokines in healthy donors and HCC patients.**
(DOC)

Table S3 **Effects of TACE on circulating lymphocyte subsets in stage III HCC patients who underwent TACE.**
(DOC)
