# Supplementary figures and images for: Increased Circulating Th17 Cells after Transarterial Chemoembolization Correlate with Improved Survival in Stage III Hepatocellular Carcinoma: A Prospective Study
Source: PLoS One. 2013 Apr 2;8(4):e60444. doi: 10.1371/journal.pone.0060444 (PMC3614950; doi:10.1371/journal.pone.0060444)

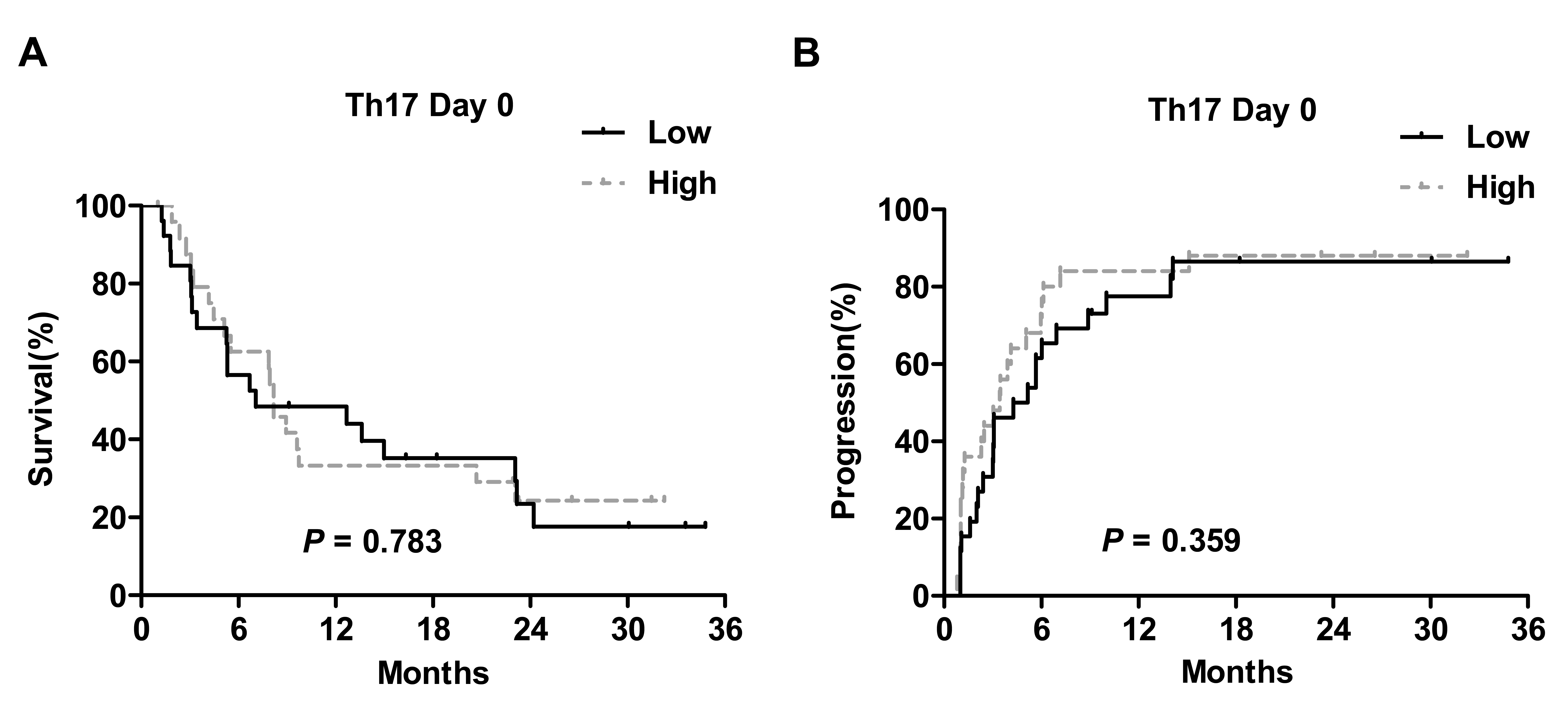

Supplement: Figure S1 — Prognostic significance of Th17 cells in HCC patients before TACE. Kaplan-Meier curve for overall survival (A) and time to progression (B) by the frequency of Th17 cells before TACE (n = 51). (TIF) [file pone.0060444.s001.tif]
