# Supplementary material for: Increased Circulating Th17 Cells after Transarterial Chemoembolization Correlate with Improved Survival in Stage III Hepatocellular Carcinoma: A Prospective Study
Source: PLoS One. 2013 Apr 2;8(4):e60444. doi: 10.1371/journal.pone.0060444 (PMC3614950; doi:10.1371/journal.pone.0060444)
Supplement: Table S1 — Comparison of the frequencies of various lymphocyte subsets in healthy donors and HCC patients. (DOC) [file pone.0060444.s002.doc]

**Table S1.** Comparison of the frequencies of various lymphocyte subsets in healthy donors and HCC patients

| Variables | HD (a) | Stage I HCC (b) | Stage III HCC (c) | *P* value | | |
| --- | --- | --- | --- | --- | --- | --- |
|  |  |  |  | b vs. a | c vs. a | c vs. b |
| CD3^+^/LYM% | 61.05±9.69 | 58.68±10.66 | 55.91±16.27 | 0.439 | 0.351 | 0.790 |
| CD4^+^/CD3^+^% | 57.33±11.60 | 55.20±9.72 | 55.42±13.18 | 0.587 | 0.779 | 0.583 |
| CD8^+^/ CD3^+^% | 35.90±11.16 | 37.39±9.98 | 37.25±11.62 | 0.867 | 0.843 | 0.992 |
| CD3^-^CD56^+^/LYM% | 20.71±7.96 | 20.51±10.24 | 18.77±10.67 | 0.950 | 0.215 | 0.412 |
| CD3^+^CD56^+^/LYM% | 2.78±1.59 | 3.66±2.91 | 3.18±3.47 | 0.645 | 0.514 | 0.277 |
| CD25^+^Foxp3^+^/CD4^+^% | 4.65±1.68 | 7.02±1.67 | 7.64±3.16 | **0.001** | **0.001** | 0.697 |
| IL-17^+^/CD4^+^% | 0.53±0.33 | 0.70±0.43 | 1.01±0.56 | 0.195 | **0.001** | **0.015** |
| IFN-γ^+^/CD4^+^% | 9.36±3.99 | 14.51±8.72 | 13.09±5.10 | 0.060 | **0.003** | 0.929 |
| IFN-γ^+^/CD8^+^% | 29.77±17.81 | 44.30±21.47 | 31.03±15.07 | **0.025** | 0.523 | **0.007** |

Abbreviations: LYM, lymphocytes; NC, HD, healthy donors. The results represent mean ± SD.
