# Supplementary material for: Increased Circulating Th17 Cells after Transarterial Chemoembolization Correlate with Improved Survival in Stage III Hepatocellular Carcinoma: A Prospective Study
Source: PLoS One. 2013 Apr 2;8(4):e60444. doi: 10.1371/journal.pone.0060444 (PMC3614950; doi:10.1371/journal.pone.0060444)
Supplement: Table S2 — Plasma levels of Th1, Th2 and Th17 associated cytokines in healthy donors and HCC patients. (DOC) [file pone.0060444.s003.doc]

**Table S2.** Plasma levels of Th1, Th2 and Th17 associated cytokines in healthy donors and HCC patients

| Cytokines | HD | | Stage I HCC | | Stage III HCC | |
| --- | --- | --- | --- | --- | --- | --- |
|  | Positive/ Total (%) | Mean pg/ml | Positive/ Total (%) | Mean pg/ml | Positive/ Total (%) | Mean pg/ml |
| IL-2 | 0/20 (0) | 0 | 6/28 (21) | 0.33 | 11/51 (22) | 0.30 |
| IL-4 | 0/20 (0) | 0 | 1/28 (4) | 0.20 | 10/51 (20) | 0.30 |
| IL-6 | 10/20 (50) | 0.74 | 20/28 (71) | 1.81 | 46/51 (90) | 7.57 |
| IL-10 | 6/20 (33) | 0.44 | 1/28 (4) | 0.08 | 11/51 (22) | 1.03 |
| IL-17 | 0/20 (0) | 0 | 0/28 (0) | 0 | 1/51 (2) | 0.49 |
| IFN-γ | 0/20 (0) | 0 | 0/28 (0) | 0 | 1/51 (2) | 0.04 |
| TNF-α | 0/20 (0) | 0 | 0/28 (0) | 0 | 3/51 (6) | 0.45 |

Abbreviations: HD, healthy donors.
