# Supplementary material for: Increased Circulating Th17 Cells after Transarterial Chemoembolization Correlate with Improved Survival in Stage III Hepatocellular Carcinoma: A Prospective Study
Source: PLoS One. 2013 Apr 2;8(4):e60444. doi: 10.1371/journal.pone.0060444 (PMC3614950; doi:10.1371/journal.pone.0060444)
Supplement: Table S3 — Effects of TACE on circulating lymphocyte subsets in stage III HCC patients who underwent TACE. (DOC) [file pone.0060444.s004.doc]

**Table S3.** Effects of TACE on circulating lymphocyte subsets in stage III HCC patients who underwent TACE

| Variables | D_0_ | D_30_ | *P* value |
| --- | --- | --- | --- |
|  |  |  | D_30_ vs. D_0_ |
| CD3^+^/LYM% | 57.22±16.36 | 58.79±14.93 | 0.992 |
| CD4^+^/ CD3^+^% | 55.12±13.49 | 51.41±11.97 | **0.020** |
| CD8^+^/ CD3^+^% | 37.15±12.02 | 39.24±10.23 | **0.015** |
| CD3^-^CD56^+^/LYM% | 19.06±11.25 | 18.86±9.08 | 0.959 |
| CD3^+^CD56^+^/LYM% | 3.58±4.20 | 4.46±4.55 | **0.007** |
| CD25^+^FOXP3^+^/CD4^+^% | 7.36±3.66 | 6.69±2.14 | 0.221 |
| IL-17^+^/CD4^+^% | 1.00±0.58 | 1.38±0.92 | **0.036** |
| IFN-γ^+^/CD4^+^% | 12.30±5.09 | 13.51±6.05 | 0.206 |
| IFN-γ^+^/CD8^+^% | 29.92±16.00 | 34.60±17.73 | **0.045** |

Abbreviations: LYM, lymphocytes; D_0_, before treatment; D_30_, 30 days after treatment. The results represent mean ± SD.
